# Assessing the Effects of Human Activities on Terrestrial Net Primary Productivity of Grasslands in Typical Ecologically Fragile Areas

**DOI:** 10.3390/biology12010038

**Published:** 2022-12-25

**Authors:** Qing Huang, Fangyi Zhang, Qian Zhang, Yunxiang Jin, Xuehe Lu, Xiaoqing Li, Jia Liu

**Affiliations:** 1 School of Environmental Science, Nanjing Xiaozhuang University, Nanjing 211171, China; 2 School of Public Administration, Nanjing University of Finance and Economics, Nanjing 210023, China; 3 School of Geomatics Science and Technology, Nanjing Tech University, Nanjing 211816, China; 4 State Key Laboratory of Soil Erosion and Dryland Farming on the Loess Plateau, Institute of Soil and Water Conservation, Northwest A&F University, Yangling 712100, China; 5 Institute of Agricultural Resources and Regional Planning, Chinese Academy of Agriculture Sciences, Beijing 100081, China; 6 School of Geography Science and Geomatics Engineering, Suzhou University of Science and Technology, Suzhou 215009, China

**Keywords:** human activities, net primary productivity, grassland degradation, climate change, forage harvest

## Abstract

**Simple Summary:**

Human activities have been found to be the major factor driving the changes in terrestrial ecosystems. However, large uncertainties remain in assessing the impact of human activities on terrestrial ecosystems. In consequence, we established a quantitative indicator based on terrestrial potential and actual net primary productivity (NPP), as well as the forage harvest NPP, to better reveal the spatiotemporal changes of grassland ecosystems in response to human activities in eastern Inner Mongolia, and to further explore the relationship between human activities and grassland degradation in typical ecologically fragile areas. The quantitative indicator showed that NPP loss induced by human activities is weakening in the study area, and a positive relationship between human activities and grassland degradation was found. The results of this study will provide a deeper and more comprehensive understanding of the grassland degradation influenced by human activities, and a method reference for similar research.

**Abstract:**

Global enhanced human activities have deeply influenced grassland ecosystems. Quantifying the impact of human activities on grasslands is crucial to understanding the grassland dynamic change mechanism, such as grassland degradation, and to establishing ecosystem protection measures. In this study, potential net primary productivity (PNPP), actual NPP (ANPP), and the forage harvest NPP (HNPP) were employed to establish the human activities index (HAI) to reveal the spatiotemporal changes of the effects of human activities on grassland ecosystems in eastern Inner Mongolia from 2000 to 2017, and to further explore the relationship between human activities and grassland degradation. The results showed that the total average PNPP, ANPP, and HNPP of grasslands in eastern Inner Mongolia were 187.2 Tg C yr^−1^, 152.3 Tg C yr^−1^, and 8.9 Tg C yr^−1^, respectively, during the period of 2000 to 2017. The HAI exhibited a clear decreasing trend during the study period, with annual mean values ranging from 0.75 to 0.47, which indicates that the NPP loss induced by human activities is weakening, and this trend is dominated by the difference between potential NPP and actual NPP. About 42.4% of the study area was non-degraded grassland, and the declining grassland degradation index (GDI) indicated that the degradation grade in eastern Inner Mongolia improved from moderate to light degradation. A positive relationship was found between HAI and GDI. This relationship was more significant in Xilingol League, which is a typical ecologically fragile area, than that in Xing’an League and Hulunbuir City.

## 1. Introduction

Net primary productivity (NPP) quantifies the total amount of net carbon fixed by plants through photosynthesis. NPP is the key component of the global carbon cycle [1], and provides a measurable and unified boundary for terrestrial ecosystems [2]. Now, it is identified as a key variable to indicating the change in ecosystem function and vegetation dynamics [3], and is widely used in the study of biodiversity [4] and environmental degradation, such as desertification [5] and grassland degradation [6,7]. Numerous studies have found that human activities have become the major factor driving the changes in terrestrial NPP [7,8,9], and NPP has been used as an indicator to assess the impact of human activities on terrestrial ecosystems [10,11,12].

Most of the studies have used the difference between potential NPP and actual NPP to assess the impact of human activities on terrestrial ecosystems [13,14,15]. The potential NPP (PNPP) is the terrestrial NPP of potential vegetation, assuming that the growth conditions of vegetation are affected only by climate change without interference from human activities. In addition, the actual NPP (ANPP) is the terrestrial NPP of the actual situation of vegetation considering the combined effects of climate change and human activities. For instance, the difference between PNPP and ANPP was employed to estimate the NPP loss in the process of global land degradation caused by human activities [13], and also to assess the situation of grassland degradation [14,15] and desertification [5]. Furthermore, the slope trends of ANPP and PNPP and their difference were used to determine the relative impacts of climate change and human activities on terrestrial dynamics, as well as to assess the progress of desertification [16] and vegetation degradation [17]. In addition, their wide applications in Qinghai–Tibet Plateau [5], Shiyang River Basin [18], Heihe Basin [19], and Northwest China [20] provided useful references for analyzing the adaptability of these methods in different regions.

However, the above methods only considered the changes in terrestrial PNPP and ANPP, while the further impacts of human activities such as grazing and crop harvest on the terrestrial ANPP were ignored. Some researchers [21,22,23] have proposed that the terrestrial NPP loss from human harvests is also a part of the impact of human activities on the terrestrial ecosystems, and the total amount of human impact on the terrestrial NPP is the sum of the difference between the PNPP and ANPP as well as the impact of human harvests. For instance, grazing or mowing for hay and silage can induce NPP loss on grassland, which is similar to harvesting for rice, wheat, and other grain crops and economic crops on cropland. It is necessary to establish a composite indicator by combining the difference between ANPP and PNPP and the impact of human harvests. Therefore, an indicator named the human activities index (HAI) was developed in this study to quantitatively assess the impact of human activities on terrestrial net primary productivity.

Grasslands are one of the most important terrestrial ecosystems, covering approximately 25% of the global terrestrial surface and holding nearly 20% of the global carbon storage [24,25]. It is sensitively fragile and susceptible to human activities [26]. Global warming and human activities, such as overgrazing and land use change, have induced widespread grassland degradation over recent decades [5,7,14]. Grassland degradation is related to the problems of declining productivity, biodiversity loss, and changing vegetation cover, and is usually monitored by these factors. Among them, the vegetation cover was found to have a significant linear correlation with the Normalized Difference Vegetation Index (NDVI) [27], and the grassland degradation index (GDI) based on vegetation cover was regarded as one of the widely used and effective factors to monitor the condition of grassland degradation [28,29]. As mentioned above, some studies employed the PNPP and ANPP for the analysis of the impact of human activities on grassland degradation [14,15,17]; however, the quantitative relationship between human activities (HAI) and grassland degradation (GDI) need to be further researched.

As we know, grasslands are the dominant terrestrial ecosystems in Inner Mongolia and also an important part of China’s northern ecological security barrier. In this study, the HAI was quantified to analyze the spatiotemporal trends of human activities on grassland NPP from 2000 to 2017 in eastern Inner Mongolia, and the GDI index was calculated to obtain the distribution of grassland degradation. Moreover, further discussion about the relationship between the HAI and GDI was performed in eastern Inner Mongolia. This study will provide a deeper understanding of the grassland degradation influenced by human activities, and will also provide a theoretical basis for grassland resource management and sustainable development in Inner Mongolia.

## 2. Materials and Methods

### 2.1. Study Area

This study was conducted in eastern Inner Mongolia, which comprises Xilingol League, Xing’an League, and Hulunbuir City. It ranges from an eastern longitude of 110°55′ to 126°23′, and a northern latitude of 41°48′ to 53°02′, and is bounded by Mongolia and Russia to the north, and adjacent to Heilongjiang, Jilin, and Hebei province to the east, with a total area of 51.54 million hectares and a resident population of 4.74 million in 2021.

Over 50% of this region is covered by grasslands, making it the most typical grassland distribution area in China. This region is one of the largest and most important pastures in China, and the continuous growth of the economy and population have promoted increasing consumption of resources, such as meat, milk, and so on. Increasing human activities, including overgrazing, and excessive land use have a significant impact on terrestrial ecosystems of this region. As showed by the Inner Mongolia statistical yearbook (http://tj.nmg.gov.cn/datashow/index.htm (accessed on 30 June 2022)), livestock yield increased from 67.2 thousand to 101.3 thousand heads during 2000–2017.

**Figure 1:**
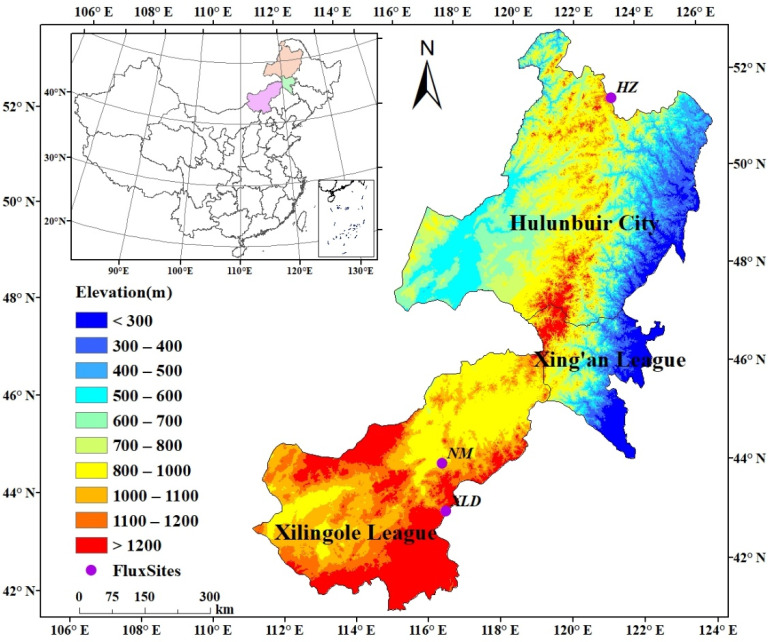
The geographical location and topography of eastern Inner Mongolia.

This region is located in the temperate continental monsoon climate zone, which is characterized by rain and heat in summer, and dry and cold in winter. The precipitation is mainly concentrated from June to September, accounting for 70% of the total amount of annual precipitation. The Greater Khingan Mountains cut through Inner Mongolia from northeast to southwest, representing a natural dividing line. As Figure 1 showed, in the east of the Greater Khingan Mountains, the hills and plains are widely distributed with elevations ranging from 300 to 600 m, ascending from east to west. This area is a semi-humid region, with an annual average precipitation from 500 to 800 mm. The west of the Greater Khingan Mountains, dominated by grasslands, is a typical semi-arid region, with an altitude of about 1000–1100 m and precipitation of 300–500 mm. Eastern Inner Mongolia is one of the main pastures in China, where the terrestrial ecosystems are greatly affected by human activities.

### 2.2. Data Sources and Processing

The dataset used in this study included Boreal Ecosystems Productivity Simulator (BEPS)-driven data, agricultural statistical data, NDVI data, and land use and land cover data.

**Figure 2:**
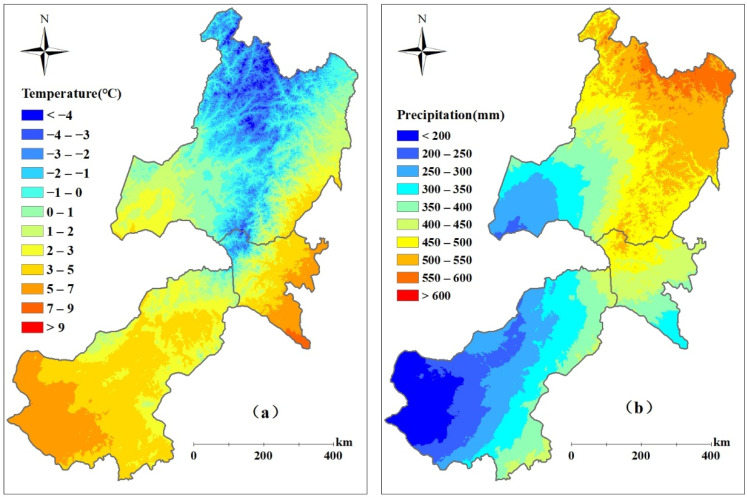
Spatial distribution of annually averaged temperature (**a**) and precipitation (**b**) during 2000–2017 in eastern Inner Mongolia.

Climate forcing data from the year 2000 to 2017 for driving the BEPS model include air temperature, precipitation, relative humidity, surface downward shortwave radiation, and wind speed. These meteorological observing station data were obtained from the China meteorological data service center (http://data.cma.cn/ (accessed on 2 November 2020)), and were used to generate the 500 m meteorological fields by means of the weighted inverse distance. In the interpolation of temperatures, a lapse rate of 0.6 °C per 100 m was assumed. The pattern of the multi-year average temperature and precipitation over eastern Inner Mongolia is shown in Figure 2. Overall, the average temperature and precipitation increase from southwest to northeast, and range from 0.45℃ to 2.9℃ and 262 mm to 535 mm, respectively.

Besides climate datasets, the leaf area index (LAI) and land cover data are also needed. The long-term LAI used in this study was generated from the surface reflectance datasets (MOD09A1) of the Moderate Resolution Imaging Spectroradiometer (MODIS) by driving an LAI inversion algorithm based on a four-scale geometrical optical model [30,31,32]. The yearly land cover data were obtained from the 500 m MODIS land cover data (MCD 12Q1), and reclassified into eight vegetation cover types according to the land cover classification scheme of the International Geosphere Biosphere Programme (IGBP). For the initialization of BEPS, the fractions of clay, silt, and sand in the soil are necessary for determining the physical parameters of the soil, including the wilting point, field capacity, porosity, hydrological conductance, and so on. These characteristics of the soil were retrieved from the harmonized global soil database (http://www.fao.org/, accessed on 1 October 2022). The MODIS 500 m NDVI data (MOD13A1) were obtained from the NASA Distributed Active Archive Center (DAAC), and the yearly MOD13A1 datasets are 16-day composites using the maximum value composite (MVC) method. In addition, the numbers of livestock data in the study area were taken from the Inner Mongolia statistical yearbook (http://tj.nmg.gov.cn/datashow/index.htm, accessed on 1 October 2022).

### 2.3. ActualNPP and PotentialNPP

#### 2.3.1. BEPS Model

The BEPS model is employed to simulate the actual NPP (ANPP) of eastern Inner Mongolia. This model is a two-leaf (sunlit and shaded leaves) model which was developed for boreal ecosystems originally, and has been improved for global carbon cycle simulations [33,34]. Eight plant functional types are defined in the BEPS model to simulate photosynthesis based on an enzyme kinetic FvCB (Farquhar, von Caemmerer, and Berry) model [35], and the actual NPP is calculated as the difference between the GPP and autotrophic respiration. Based on previous studies, the BEPS is capable of simulating both GPPs and NPPs for a wide variety of terrestrial ecosystems at the site and global scale [36,37,38].

#### 2.3.2. Miami Model

The Miami model is used to simulate the potential NPP (PNPP) of eastern Inner Mongolia. This model is an empirical model, and the PNPP in a particular location is calculated as the minimum of the temperature and precipitation regression functions [39]. As we know, the adaptability of the empirical method is affected by different ecosystem structures and climate conditions [40]. The parameters of the classical Miami model were calibrated by fitting the NPP observations with temperature and precipitation data in the early years before the 1970s and must be re-parameterized with new sets of productivity and climate change observations; Zaks [41] collected 3023 global NPP field observations and corresponding meteorological data to calibrate the parameters and develop several statistic models. We chose the new calibrated Miami model to calculate the PNPP in this study.

### 2.4. Forage Harvest NPP

Forage harvest NPP (HNPP) is the consumed grass biomass by grazing or mowing for hay and silage, which is regarded as one of the human activities affecting the terrestrial ecosystems [21]. HNPP was estimated as the difference between livestock feed demand and feed supply. Among them, feed demand was calculated separately for six livestock species, i.e., cattle and buffaloes, goats and sheep, horses, asses, mules, and camels. The yearly consumed forage was estimated by using species–specific data for daily feed intake, and each livestock type was converted into standard sheep units outlined in Table 1 based on the China Agricultural Industry Standard NY/T 635-2015. The livestock was divided into young livestock and adult livestock in this study, and the coefficient of adult sheep was 1. One sheep unit needed 1.8 kg of dry-matter coarse forage per day. The feed supply consists of crop residues used for feed and other market feeds. Due to the lack of robust data on commercial or supplementary feeds, we assumed that the feed supply of grassland was equal to the crop residues used as feed. The conversion rate of crop residues to feed was assumed to be 25% [42]. The HNPP was spatially allocated to the grasslands by calculating the HNPP as a proportion of the ANPP for each grazing land class as described in Haberl [21].

### 2.5. Human Activities Index

To assess the impact of human activities on grasslands, a human activities index (HAI) referenced from [22] was developed based on the ANPP, PNPP, and HNPP, according to Equation (1). The difference between the PNPP and ANPP was considered as terrestrial NPP changes resulting from land conversion and land use by human activities. In addition, the HNPP was the terrestrial NPP harvested by humans. A negative HAI suggested that there is a positive effect of human activities such as ecological protection and management on terrestrial NPP, and a positive HAI represented an NPP loss induced by human activities.

(1) $$HAI=\frac{PNPP-ANPP+HNPP}{PNPP}$$

### 2.6. Grassland Degradation Index

The grassland degradation index (GDI) [28] was calculated to represent the status of grassland degradation as Equation (2). The GDI values were graded into four classes of degradation, i.e., seriously degraded (GDI > 3.0), moderately degraded (2.0 < GDI ≤ 3.0), lightly degraded (1.0 < GDI ≤ 2.0), and not degraded (GDI ≤ 1.0).

(2) $$GDI=\frac{\sum_{i=1}^{4}D_{i}\times A_{i}}{A}$$

where A_i_ is the distribution area of level i, and A is the total area of grasslands. D_i_ is the level of the degradation class depending on the vegetation cover (V_c_), and D_i_ ranges from 1 to 4. According to the significant linear correlation between the V_c_ and NDVI found by [27], the V_c_ is calculated as follows:

(3) $$Vc=\frac{NDVI-NDVI_{\min}}{NDVI_{\max}-NDVI_{\min}}$$

where NDVI_min_ is the minimal NDVI value for bare soil, NDVI_max_ is the maximal NDVI value for pure vegetation of the grasslands, and NDVI is the located NDVI value. V_c_ is classified into four classes, and the D_i_ is assigned to be 1 to 4 when the V_c_ value is larger than 0.85, between 0.65 and 0.85, between 0.25 and 0.60, and less than 0.25, respectively.

## 3. Results

### 3.1. Actual NPP Simulated by BEPS

**Figure 3:**
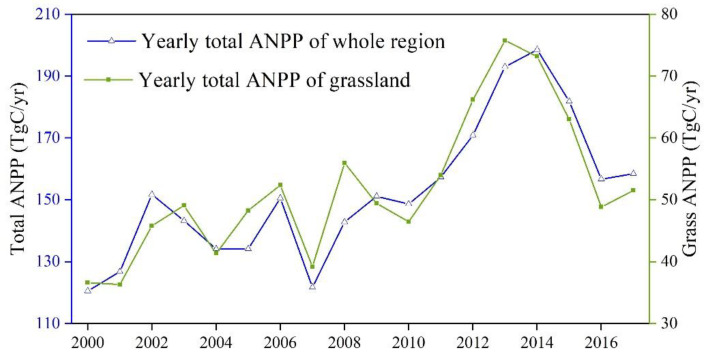
The variation in the total ANPP (blue) and total grasslands NPP (green) of eastern Inner Mon-golia from 2000 to 2017.

The total actual NPP (ANPP) simulated by the BEPS model in eastern Inner Mongolia ranged from 120.6 Tg C yr^−1^ to 198.5 Tg C yr^−1^(1 Tg = 10^12^ g), with an average of 152.3 Tg C yr^−1^ over the period of 2000–2017 (Figure 3), and increased at a rate of 3.1 Tg C yr^−1^ since 2000. The grasslands’ total ANPP ranged from 36.3 Tg C yr^−1^ to 75.6 Tg C yr^−1^, with an average of 51.9 Tg C yr^−1^. This ANPP of the grasslands took about 34.1% of the total ANPP in eastern Inner Mongolia, where the grasslands, however, covers 64.9% of the area.

The total ANPP of the grasslands varied significantly in Xilingol League, Xing’an League, and Hulunbuir City, and the average value of the above three regions were 19.9 Tg C yr^−1^, 12.2 Tg C yr^−1^, and 19.7 Tg C yr^−1^, accounting for 96.9%, 68.9%, and 17.3% of the total vegetation ANPP in the corresponding regions, respectively. With over 50% forest coverage, the proportion of the grasslands ANPP to the total vegetation ANPP in Hulunbuir City is the smallest among the three regions.

**Figure 4:**
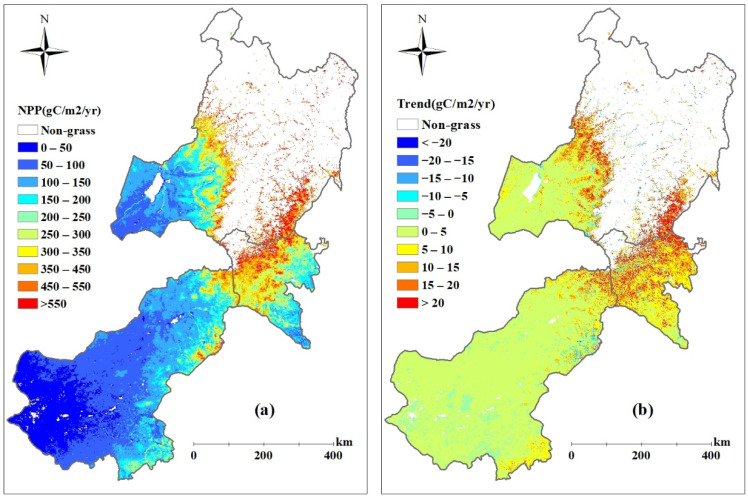
Spatial distribution of simulated averaged ANPP (**a**) and its trends (**b**) over the period of 2000 to 2017 in eastern Inner Mongolia.

The average annual ANPP of the grasslands during 2000–2017 in eastern Inner Mongolia is shown in Figure 4a. The annual ANPP showed a gradient increasing from southwest to northeast, and the values were mostly below 200 g C m^−2^ yr^−1^ in extensive areas of eastern Inner Mongolia. The annual ANPP of he grasslands in Xilingol League ranged from 74.5 to 146.1 g C m^2^ yr^−1^ during 2000–2017, with an average of 102.5 g C m^2^ yr^−1^, which was obviously smaller than that of Xing’an League (292.4 g C m^2^ yr^−1^) and Hulunbuir City (242.1 g C m^2^ yr^−1^)during the same period. As shown in Figure 4b, the annual ANPP showed an increasing trend in most areas of eastern Inner Mongolia, and the average increase rates were 2.3 g C m^2^ yr^−1^, 11.6 g C m^2^ yr^−1^, and 5.7 g C m^2^ yr^−1^ in Xilingol League, Xing’an League, and Hulunbuir City, respectively.

### 3.2. Potential NPP Calculated by Miami Model

**Figure 5:**
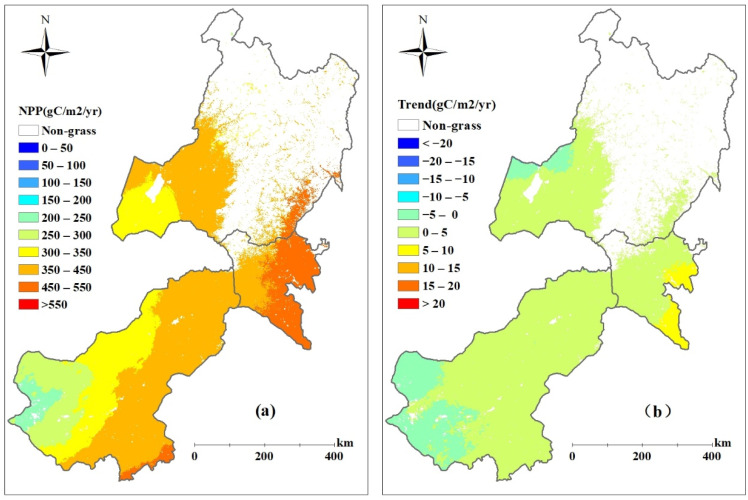
Spatial distribution of simulated averaged PNPP (**a**) and its trends (**b**) over the period of 2000 to 2017 in eastern Inner Mongolia.

The total potential NPP (PNPP) calculated by the Miami model in eastern Inner Mongolia ranged from 163.5 Tg C yr^−1^ to 210.2 Tg C yr^−1^(1 Tg = 10^12^ g) during the period of 2000–2017, with an average of 187.2 Tg C yr^−1^. The total PNPP of the grasslands changed with fluctuations from 99.6 Tg C yr^−1^ to 136.3 Tg C yr^−1^ in the same period, with an average of 120.5 Tg C yr^−1^, accounting for 64.3% of the total PNPP. The multi-year (2000–2017) averaged annual PNPP showed a gradient increasing from the west to the east (Figure 5a), which ranged from 74.5 to 146.1 g C m^2^ yr^−1^. Compared with Xilingol League and Hulunbuir City, the more abundant precipitation and warmer temperature in Xing’an League (Figure 2) makes the PNPP there larger than that in other regions. Meanwhile, as shown in Figure 5b, the average PNPP showed a slight increase trend in most areas of eastern Inner Mongolia except the west of Xilingol League.

### 3.3. Forage Harvest NPP Based on the Feed–Supply Balance

**Figure 6:**
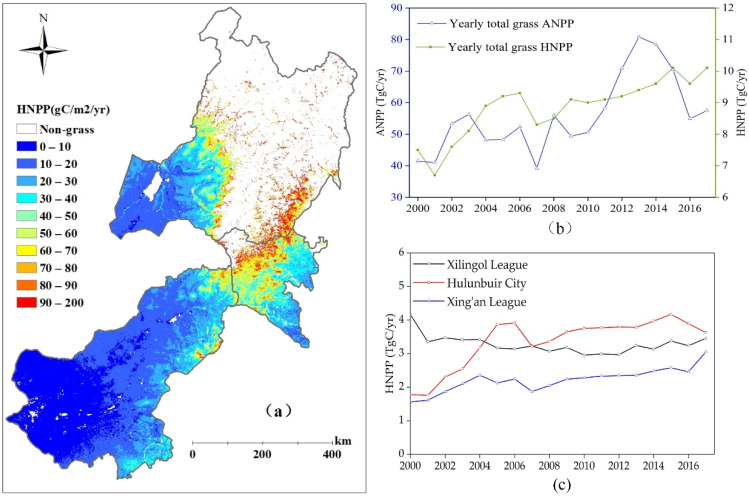
Spatial distribution of calculated averaged HNPP (**a**), variation of total grasslands ANPP and HNPP (**b**), and variation of total grasslands HNPP in Xilingol League, Hulunbuir City, and Xing’an League (**c**) over the period of 2000 to 2017 in eastern Inner Mongolia.

The biomass harvest from the grasslands, defined as forage harvest NPP (HNPP), was estimated to be in the range of 6.7–10.2 Tg C yr^−1^ over the period of 2000 to 2017. The average total HNPP was 8.9 Tg C yr^−1^, accounting for about 17.1% of the total grasslands ANPP. As shown in Figure 6c, the total grasslands HNPP in Hulunbuir City and Xing’an League presented a clear increasing trend, and the average values were 3.4 Tg C yr^−1^ and 2.2 Tg C yr^−1^ in these two regions, respectively. However, the trend in Xinan League was not pronounced, and its average value was 3.3 Tg C yr^−1^. In most areas of eastern Inner Mongolia, the average annual HNPP was less than 20 g C m^2^ yr^−1^, and as shown in Figure 6a, the average HNPP increased from west to east.

### 3.4. Quantitative Assessment of the Impact of Human Activities on Terrestrial NPP in Eastern Inner Mongolia

**Figure 7:**
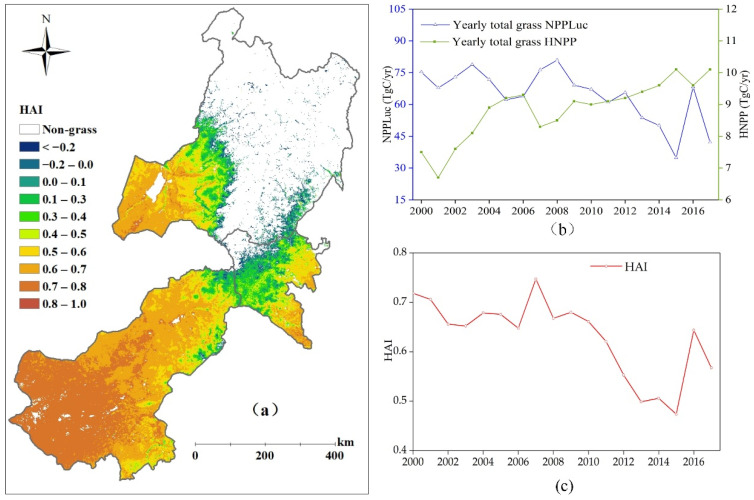
Spatial distribution of calculated multi-year averaged HAI (**a**), variation of total grasslands NPP_Luc_ and HNPP (**b**), and variation of annual mean HAI (**c**) over the period of 2000 to 2017 in eastern Inner Mongolia.

The HAI was divided into two parts to better analyze its changing trends. One was the difference between the PNPP and ANPP, defined as NPP_Luc_, which denotes the impact of human-induced land conversions on terrestrial NPP. The other was the HNPP. As shown in Figure 7c, the annual mean HAI changed with fluctuations from 0.47 to 0.75 during 2000–2017, with an average of 0.63, and the HAI exhibited a clear decreasing trend especially in the period of 2007–2015. The NPP_Luc_ of the grasslands was significantly larger than the HNPP, ranging from 80.9 Tg C yr^−1^ to 34.8 Tg C yr^−1^, while the total HNPP changed from 6.7 Tg C yr^−1^ to 10.1 Tg C yr^−1^ over the period of 2000–2017. Thus, it is obvious that the changes in HAI was mainly due to the NPP_Luc_ in eastern Inner Mongolia. As shown in Figure 7b, a clear decreasing trend of the NPP_Luc_, which was similar to that of the HAI, was presented and compared with the gradually increasing trends of the HNPP. The multi-year averaged HAI increased from southwest to northeast (Figure 7a). Due to the low ANPP, the HAI in most areas of the southwest was larger than 0.7. Nevertheless, there were many points scattered in the Greater Khingan Mountains with HAI values less than 0, where the ANPP was significantly larger than thet PNPP because of the misclassification of the forests as grasslands.

### 3.5. Quantitative Assessment of Grassland Degradation in Eastern Inner Mongolia

The annual mean GDI changes with fluctuations from 2.29 to 1.61 in eastern Inner Mongolia from 2000 to 2017, and presents a weak decreasing trend, which indicates that the grassland degradation situation of the whole region is changing from moderately degraded to lightly degraded. The non-degraded grasslands accounted for about 42.4% of the total area, while the area of lightly degraded, moderately, and seriously degraded grasslands were about 22.0%, 28.8, and 6.8% of the total area respectively. The yearly percentages of different degradation levels are shown in Table S1.

**Figure 8:**
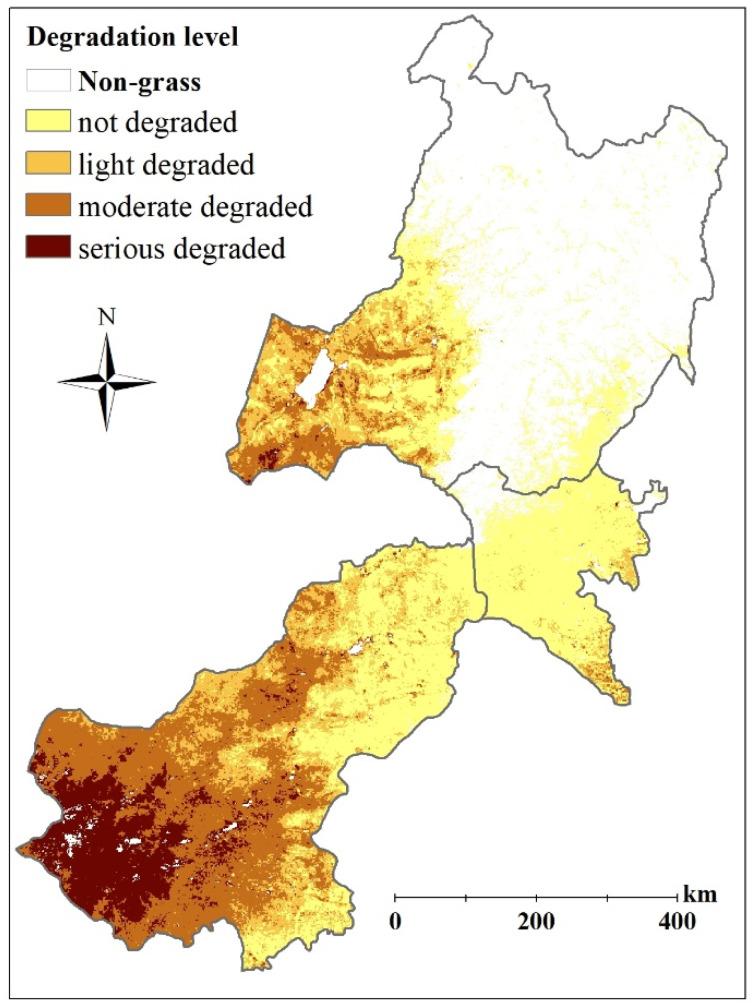
Spatial distribution of grassland degradation in the year of 2017 in eastern Inner Mongolia.

The annual mean GDI in Xilingol League, Xing’an League, and Hulunbuir City were 2.41, 1.18, and 1.50, respectively. The situation of grassland degradation was most serious in Xilingol League, where the seriously and moderately degraded grasslands accounted for about 11.0% and 41.9% of the total area, respectively. Nevertheless, seriously degraded grasslands have little distribution in the other two regions, with proportions less than 1.0%. The situations of grassland degradation in Xing’an League and Hulunbuir City were close to the slight degradation grade, with the slightest degradation in Xing’an League, where 84.9% of the area is at the non-degradation level, and 12.3% was at the slight degradation level. While the grassland degradation in Hulunbuir City was slightly more severe than that in Xing’an League, areas of moderately and lightly degraded grasslands accounted for about 12.1% and 23.4% of its total area, respectively. The spatial distribution of grassland degradation in the year 2017 is shown in Figure 8.

## 4. Discussion

### 4.1. Uncertainty in the Calculation of the Human Activity Index

The HAI index is calculated by using the variables ANPP, PNPP, and HNPP in this study. Numerous studies used only the difference between the PNPP and ANPP to assess the impact of human activities on terrestrial NPP [5,16,20]. However, the HNPP sometimes was ignored and should be considered as one part of the HAI [21,22]. The HAI is a comparable indicator in different countries and regions, as shown in Table 2. According to our results, the HAI decreased from 75% to 47% from 2000 to 2017 in the grasslands, which is larger than that in different countries. Previous studies have suggested that the HAI in forest areas is low, as forest areas suffered less disturbance than other land cover types. In contrast, the HAI on cropland and grasslands sometimes is high. The high HNPP from the harvest of crops sometimes leads to a high HAI on cropland [43], and the low ANPP and high HNPP on grasslands resulting from grassland degradation and grazing sometimes lead to a large difference between the ANPP and PNPP and also a high HAI. Therefore, it is reasonable that the value of the HAI in our study is higher than that of other regions, especially with high forest coverage, and being close to the coastal areas of Jiangsu province in China, dominated by cropland [44]. In addition, in the city of Guangzhou, China, as well as the Yangtze River delta, the HAIs are also high, mainly due to the low ANPP and high PNPP in cities.

Although the HAI in this study is reasonable compared with previous studies, uncertainties still exist in our calculation and also in similar studies. For example, the difference between the ANPP and PNPP sometimes brings in errors by different model structures, i.e., the ANPP and PNPP are calculated by different kinds of models or NPP products. For example, Chen et al. [46] used the Zhouguangsheng model and MOD17A3 production to obtain the PNPP and ANPP, respectively. In this study, although the input data are the same, however, principle differences between the BEPS model (process-based ecological model) and Miami model (light energy utilization model) still bring uncertainties to the calculated results.

The annual mean ANPP of the grasslands in eastern Inner Mongolia from 2000 to 2017 ranges from 102.5 g C m^−2^ yr^−1^ to 292.4 g C m^−2^ yr^−1^, which is close to 281.3 g C m^−2^ yr^−1^, calculated by Mu [50] in the same region. Meanwhile, the ANPP was validated with the FLUX data at the sites of NM and XLD (Table 3), summarized by Yu [51]. We assumed that the NPP was half of the GPP, and PRE% in these two sites were only 17.76% and 8.13%, respectively. However, the PNPP is hard to verify using the flux data. We also assumed that the ANPP of the natural forest was equal to the PNPP [21], considering the natural forest is rarely affected by human activities in the Greater Khingan Mountains. For this reason, the PRE% of the PNPP in the flux site of HZ is only 4.76%. On the whole, our results are reasonable.

### 4.2. Drivers of the Changes of Human Activity Index in Eastern Inner Mongolia

As calculated in Section 3.4, the HAI consists of two parts, i.e., NPP_Luc_ and HNPP. The average total NPP_Luc_ and HNPP of the grasslands was 64.55 Tg C yr^−1^ and 8.85 Tg C yr^−1^. Thus, the changes of the HAI was dominated mainly by NPP_Luc_ in eastern Inner Mongolia.

The NPP_Luc_ is the difference between the PNPP and ANPP, and denotes the impact of human-induced land conversions on terrestrial NPP [21,22]. Land cover and land use changes induced by human activities, such as deforestation [52], and desertification due to overgrazing on grasslands [9,16], were considered to be the main drivers of changes in the NPP_Luc_ [21,53]. Moreover, human managements, such as ecosystem protection and irrigation, can effectively improve terrestrial ANPP. For example, irrigation in cultivated land in arid areas can reduce the water stress on crops, and significantly increase the crop yield. In this case, the irrigation land can have a higher ANPP than PNPP [21]. NPP_Luc_ changes driven by these factors can be regarded as a direct impact of human activities.

Some factors indirectly influenced by human activities can also drive the changes of NPP_Luc_. For example, the continuously increasing CO_2_ concentration and global warming, resulting from human carbon emission, were found to be of significant impact on terrestrial ANPP [54]. However, these changes, induced by indirect factors, sometimes combined the effects of climate change and human activities and are hard to distinguish, although some studies have tried to separate them by the slope trends of the ANPP and PNPP [16,17].

### 4.3. Relationship between HAI and Grassland Degradation in Eastern Inner Mongolia

**Figure 9:**
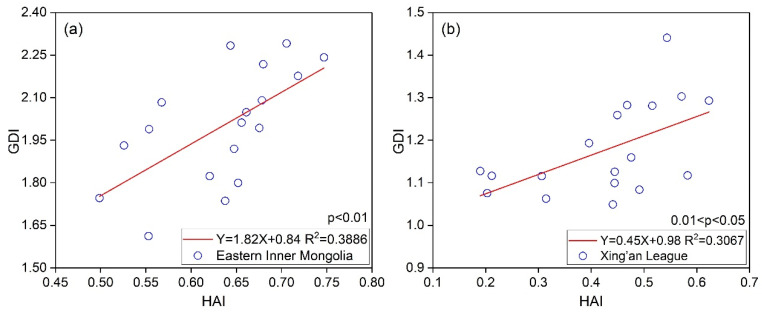

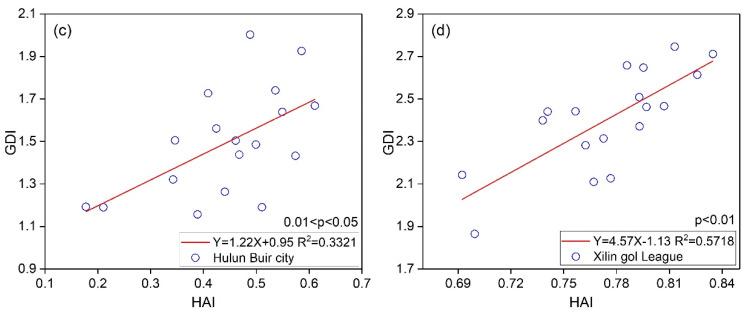
Relationship between HAI and grassland degradation in eastern Inner Mongolia (**a**), Xing’an League (**b**), Hulunbuir City (**c**), and Xilingol League (**d**).

Numerous studies have found that human activities were the main factors in driving grassland degradation by different means [5,7,54]. We established the relationship between the HAI and grassland degradation which was presented in Section 3 in eastern Inner Mongolia (Figure 9a), Xing’an League (Figure 9b), Hulunbuir City (Figure 9c), and Xilingol League (Figure 9d). The R^2^ in different areas ranges from 0.3067 to 0.5718, and the relationships are statistically significant (*p* < 0.05). The positive relationship between the HAI and GDI indicated that grassland degradation might worsen owing to the increasing intensity of human activities.

In addition, it is interesting to find that the relationship between the HAI and GDI in Xilingol League was more significant than that in the other regions. According to Figure 2, higher temperatures and less precipitation were distributed in Xilingol League, compared with the other two regions, especially in western Xilingol League. This region is a typical ecologically fragile area, and the dominant type of grassland is desert grasslands. The ANPP was low as shown in Figure 4, and the terrestrial ecosystems is more sensitive to human activities such as grazing or climate change in this region. The negative effects of human activities or climate change are more likely to cause grassland degradation. Therefore, the HAI shows a better correlation with GDI in this region. In contrast, the grasslands in Xing’an League and Hulunbuir City are mostly distributed in the transitional zone from forests to grasslands, and the ANPP in these regions is significantly larger than that in Xilingol League. Due to the stability of grassland ecosystems, the HAI is less sensitive to GDI.

As presented in Section 3.4, the HAI exhibited a clear decreasing trend during the study period. However, the increment of the HNPP calculated by the number of livestock indicates that grazing was increasing from 6.7 to 10.2 Tg C yr^−1^ in the study area. The decrease in the HAI is mainly due to the increase in the ANPP as shown in Figure 6b. Positive human activities, such as the implementation of a grasslands management policy and ecosystems protection programs, have effectively improved the grasslands ANPP and also reversed the degradation of grasslands [7].

## 5. Conclusions

In this study, the potential NPP, actual NPP, and forage harvest NPP were employed to define the HAI index to analyze the spatiotemporal trends of human activities on terrestrial NPP in a typical grasslands ecosystem in eastern Inner Mongolia, and to further explore the relationship between the HAI and grassland degradation. The results showed that the HAI exhibited a clear decreasing trend during 2000–2017, especially from 2007 to 2015, with the annual mean values ranging from 0.75 to 0.47. The decreasing trend of the HAI indicates that the NPP loss induced by human activities is weakening, and this trend is mainly due to the difference between the potential NPP and actual NPP. Among them, the total average PNPP and ANPP were 187.2 Tg C yr^−1^ and 152.3 Tg C yr^−1^, respectively, during the period of 2000 to 2017, and the difference between the averaged PNPP and ANPP was 34.9 Tg C yr^−1^, which was significantly higher than the average HNPP (8.9 Tg C yr^−1^).

Based on the GDI index, the grassland degradation situation in eastern Inner Mongolia was improved from moderately degraded to lightly degraded. The non-degraded grasslands accounted for about 42.4% of the total area, significantly larger than that of lightly degraded (22.0%), moderately (28.8%), and seriously (6.8%) degraded grasslands. A positive relationship was found between the HAI and GDI, and this relationship was more significant in Xilingol League which was a typical ecologically fragile area than that in Xing’an League and Hulunbuir City.

## Figures and Tables

**Table 1:** The coefficient of one sheep unit for livestock.

Livestock	Cow	Horse	Donkey	Mule	Camel	Sheep
Young livestock	2.5	2.5	2	2.5	3.5	0.7
Adult livestock	5	5	5	4	7	1

**Table 2:** Comparison of the HAI estimated in this study with previous studies.

Area	Period	Objects	HAI (%)	References
Global	1910–2005	All land covers	13–25 (↑)	[23]
Hungary	1961–2005	All land covers	67–49 (↓)	[43]
Spain	1955–2003	All land covers	67–61 (↓)	[45]
China	2001–2010	All land covers	49–58 (↑)	[46]
The Yangtze River Delta	2005–2015	All land covers	59–72 (↑)	[47]
Coastal Areas of Jiangsu	2000–2010	Cropland dominance	50–71 (↑)	[44]
Guangzhou *	2001–2013	All land covers	31–80 (↑)	[48]
Northeast China *	2000–2011	Marshes	6–14 (↑)	[49]
Eastern Inner Mongolia	2000–2017	Grasslands	75–47 (↓)	this study

Note: * HNPP was not considered in the study.

**Table 3:** Comparison of ANPP/PNPP with observations.

Code	Types	Period	GPP_obs_	ANPP/PNPP	RPE%
NM	Grasslands	2004–2008	231.66 ± 111.13	136.39	17.76%
XLD	Grasslands	2006	294.44	159.20	8.13%
HZ *	Forests	2010	962.75	504.29	4.76%

Note: RPE means Relative Predictive Error and is calculated as (2*ANPP − GPP_obs_)/GPP_obs_ × 100%; * value in HZ is PNPP and values in NM, XLD is ANPP.

## Data Availability

Not applicable.

## Supplementary Materials

The following are available online at https://www.mdpi.com/article/10.3390/biology12010038/s1, Table S1: Proportion of the grasslands area of different degradation levels to the total area.
