# Supplementary material for: Assessing the Effects of Human Activities on Terrestrial Net Primary Productivity of Grasslands in Typical Ecologically Fragile Areas
Source: Biology (Basel). 2022 Dec 25;12(1):38. doi: 10.3390/biology12010038 (PMC9855355; doi:10.3390/biology12010038)
Supplement: Supplementary file 1 [file biology-12-00038-s001.zip › biology-2085684-supplementary.pdf]

**Supplementary Materials**

**Table S1.** Proportion of the grasslands area of different degradation levels to the total area

| year | not degraded | light degraded | moderate degraded | serious degraded |
|---|---|---|---|---|
| 2000 | 29.65% | 26.92% | 39.58% | 3.85% |
| 2001 | 29.40% | 25.09% | 32.51% | 13.01% |
| 2002 | 40.62% | 23.75% | 29.46% | 6.18% |
| 2003 | 48.95% | 23.08% | 27.07% | 0.90% |
| 2004 | 38.83% | 20.71% | 33.04% | 7.42% |
| 2005 | 47.47% | 19.89% | 18.49% | 14.15% |
| 2006 | 43.02% | 24.26% | 30.45% | 2.28% |
| 2007 | 27.10% | 27.74% | 39.05% | 6.10% |
| 2008 | 54.59% | 20.13% | 22.32% | 2.96% |
| 2009 | 32.90% | 19.25% | 41.00% | 6.84% |
| 2010 | 37.27% | 26.70% | 29.96% | 6.08% |
| 2011 | 54.32% | 17.13% | 20.43% | 8.13% |
| 2012 | 59.32% | 20.38% | 20.07% | 0.23% |
| 2013 | 54.81% | 18.72% | 23.58% | 2.90% |
| 2014 | 50.41% | 18.96% | 17.74% | 12.90% |
| 2015 | 44.12% | 22.32% | 24.10% | 9.45% |
| 2016 | 29.86% | 19.53% | 43.01% | 7.59% |
| 2017 | 40.67% | 21.02% | 27.62% | 10.69% |
